# Toward precision in diagnosing necrotic bowel: Gold standards and weighted risk stratification

**DOI:** 10.1016/j.aicoj.2026.100056

**Published:** 2026-03-25

**Authors:** Xue Liu, Peng Liu

**Affiliations:** a Department of Anesthesiology, the First Affiliated Hospital of Dalian Medical University, Dalian, Liaoning Province, China; b Department of Heart Intensive Care Unit, the First Affiliated Hospital of Dalian Medical University, Dalian, Liaoning Province, China

To the Editor,

We read with great interest the study by Andrei et al. regarding the prediction of necrotic bowel (NB) in ICU patients [1]. This work addresses the critical challenge of distinguishing irreversible necrosis from reversible ischemia, a priority highlighted by the World Society of Emergency Surgery (WSES) guidelines [2]. However, to enhance the translational value of these findings, we wish to discuss two methodological points.

First, regarding the diagnostic “gold standard.” While the authors commendably acknowledged the lack of histopathological confirmation in their limitations, we emphasize that this omission may introduce a fundamental misclassification bias. As demonstrated by Calame et al., visual assessment during surgery can be challenging in differentiating severe nonocclusive ischemia from irreversible necrosis [3]. If the outcome variable (NB) relies solely on macroscopic judgment, it may overestimate necrosis in reversible cases. This could compromise the internal validity of the identified risk factors—for instance, biomarkers like LDH might correlate with the intensity of ischemic injury rather than the irreversibility of necrosis itself.

Second, regarding the translation of risk factors into clinical tools. The authors proposed a cumulative approach based on the number of criteria met. However, the multivariable analysis reveals vast disparities in effect sizes: the Odds Ratio for LDH (7.135) is more than double that of active fluid removal (3.148). A simple “counting criteria” approach implies equal weight for all factors, which is statistically suboptimal. We suggest that a weighted risk score derived from regression coefficients—similar to the evolution of the qSOFA score defined in the Sepsis-3 consensus [4]—would be far more clinically actionable. A weighted nomogram would provide clinicians with a precise stratification tool to guide the difficult decision of surgical exploration.

## CRediT authorship contribution statement

Xue Liu and Peng Liu conceived the idea and drafted the manuscript. Peng Liu critically revised the text for intellectual content. All authors read and approved the final manuscript.

## Declaration of Generative AI and AI-assisted technologies in the writing process

We used ChatGPT (OpenAI, San Francisco, California) solely for language polishing. All texts were subsequently reviewed, revised, and approved by the authors.

## Declaration of competing interest

The authors used Claude (Anthropic) for language editing. The authors reviewed and edited the content as needed and take full responsibility for the content of the publication.
